# Direct Observation of Defects and Increased Ion Permeability of a Membrane Induced by Structurally Disordered Cu/Zn-Superoxide Dismutase Aggregates

**DOI:** 10.1371/journal.pone.0028982

**Published:** 2011-12-28

**Authors:** Inhee Choi, Hyeon Don Song, Suseung Lee, Young In Yang, Joo Hyun Nam, Sung Joon Kim, Jung-Joon Sung, Taewook Kang, Jongheop Yi

**Affiliations:** 1 School of Chemical and Biological Engineering, Institute of Chemical Processes, Seoul National University, Seoul, Republic of Korea; 2 Department of Physiology, Seoul National University College of Medicine, Seoul, Republic of Korea; 3 Department of Neurology, Seoul National University College of Medicine, Seoul, Republic of Korea; 4 Department of Chemical and Biomolecular Engineering, Sogang University, Seoul, Republic of Korea; Russian Academy of Sciences - Institute for Biological Instrumentation, Russian Federation

## Abstract

Interactions between protein aggregates and a cellular membrane have been strongly implicated in many protein conformational diseases. However, such interactions for the case of Cu/Zn superoxide dismutase (SOD1) protein, which is related to fatal neurodegenerative disorder amyotrophic lateral sclerosis (ALS), have not been explored yet. For the first time, we report the direct observation of defect formation and increased ion permeability of a membrane induced by SOD1 aggregates using a supported lipid bilayer and membrane patches of human embryonic kidney cells as model membranes. We observed that aggregated SOD1 significantly induced the formation of defects within lipid membranes and caused the perturbation of membrane permeability, based on surface plasmon resonance spectroscopy, atomic force microscopy and electrophysiology. In the case of apo SOD1 with an unfolded structure, we found that it bound to the lipid membrane surface and slightly perturbed membrane permeability, compared to other folded proteins (holo SOD1 and bovine serum albumin). The changes in membrane integrity and permeability were found to be strongly dependent on the type of proteins and the amount of aggregates present. We expect that the findings presented herein will advance our understanding of the pathway by which structurally disordered SOD1 aggregates exert toxicity *in vivo*.

## Introduction

Cu/Zn-superoxide dismutase (SOD1) is a ubiquitous enzyme and has been linked to amyotrophic lateral sclerosis (ALS), also known as Lou Gehrig's disease [1], [2], [3]. Details of the mechanism leading to the development of neurodegeneration in ALS are currently unclear, but aberrant SOD1 aggregate formation has been strongly implicated as a causative factor in the disease. A number of pathogenic mechanisms have been proposed for the relationship between ALS and SOD1 aggregates, including the disruption of axonal transport [4], the aberrant binding of apoptosis regulators [5], inhibition of the proteasome [6], perturbations in mitochondrial function [7]. In these mechanisms, interactions between the cellular membrane and the aggregate have been somewhat overlooked, but are now thought to be a factor in other protein-aggregation disease mechanisms [8]. The disease relevance of protein-membrane interactions can be elucidated with investigating the membrane interactions of the protein aggregates to travel intercellularly [9], [10] and the toxic actions executed at the mitochondrial level through voltage-dependent anion channel [11], [12]. Therefore, a better understanding of how SOD1 aggregate interacts with a membrane and what is the nature of the consequences of such interactions might provide a mechanism that compliments the existing thinking on this subject.

**Figure 1:**
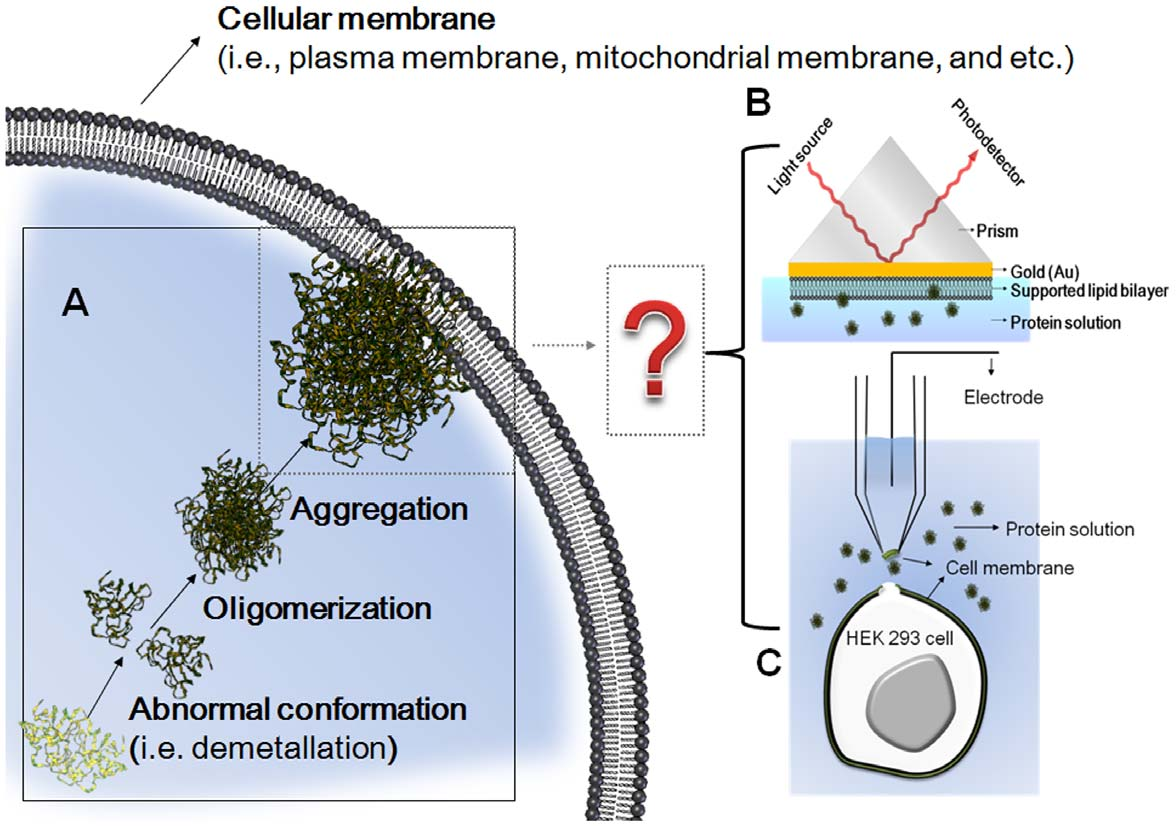
Experimental approaches for understanding the interaction between SOD1 aggregates and cellular membranes. (A) A cartoons showing the protein's aggregation and its interaction with a cellular membrane. (B) The SPR instrumental setup equipped with a flow cell used in an *in vitro* observation with an artificial SLB. (C) The inside-out configuration of the patch clamp used in an observation with a real cell membrane patch.

Moreover, the hypothesis that SOD1 variants might aggregate on mitochondrial membrane surfaces has recently been supported by the demonstration that SOD1 aggregates are attached to the cytoplasmic face of the mitochondrial membrane in transgenic ALS mice [13], [14], [15]. Considering the ubiquitous distribution of SOD1 in a cell (i.e. cytosol, nucleus, peroxisomes, lysosomes, and mitochondrial intermembrane space), it would not be surprising that SOD1 species would eventually interact with cell membranes, as shown in the scheme in Figure 1A. However, *in vivo* monitoring of protein-aggregation-associated pathogenic events is a difficult task. Therefore, *in vitro* observation of cell membrane behaviors changed by a specific type of protein, which differs from that of other normal proteins, represents a meaningful approach to understand the membrane components (i.e. lipid molecules)-mediated cytotoxic actions generated *in vivo*.

We herein, for the first time, report on such membrane-protein aggregate interactions by investigating SOD1 aggregate-induced changes in both the morphology of a supported lipid bilayer (SLB) (Figure 1B) and the ion permeability of a cellular membrane (Figure 1C). Significant morphological changes of the SLB in terms of shape and surface coverage after exposure to SOD1 aggregates were characterized by the surface plasmon resonance (SPR) spectroscopy [16] and atomic force microscopy (AFM) [17], both of which are surface sensitive label-free techniques. Since membrane damage could constitute a viable candidate for membrane permeabilization [18], [19], [20], by measuring the current changes in membrane patches detached from human embryonic kidney (HEK 293) cells, we also observed unregulated membrane permeabilization induced by aggregate-membrane interactions.

## Results

### Abnormal properties of structurally disordered SOD1

**Figure 2:**
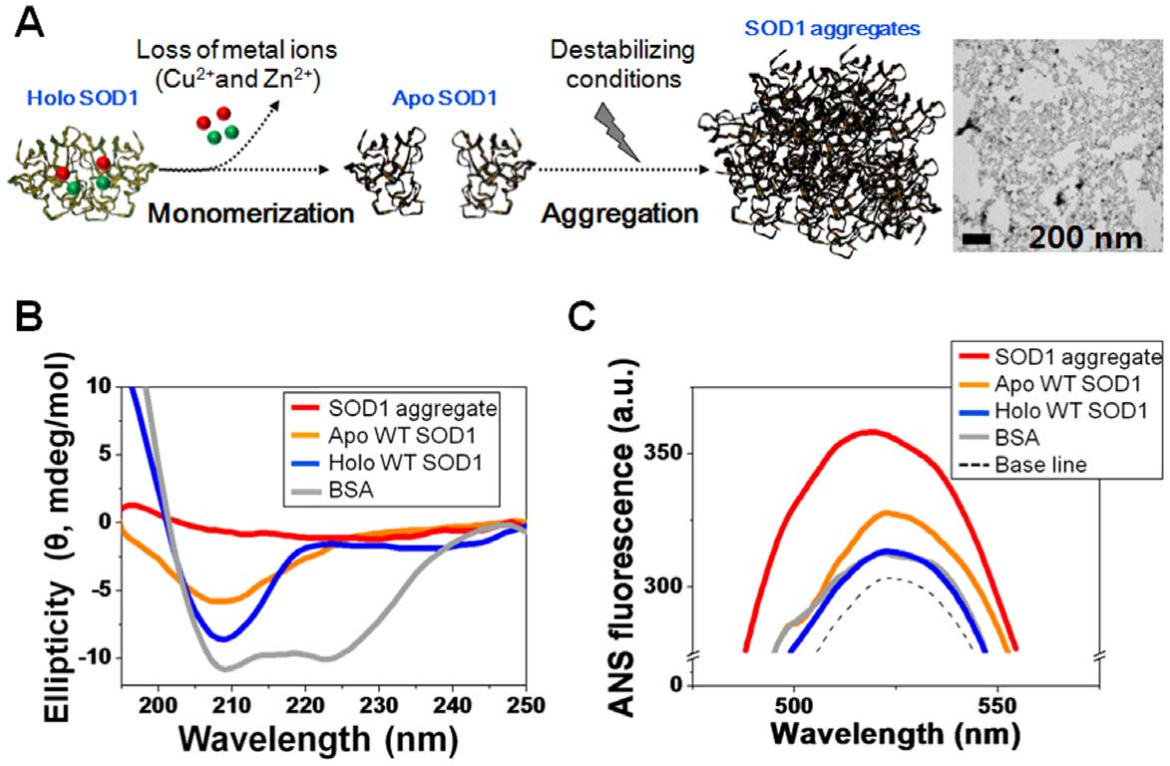
Schematic illustration of SOD1 aggregation and its characteristics. (A) A scheme for the aggregation of SOD1. Inset is a shape of as-prepared SOD1 aggregate. Bars, 200 nm. (B) CD spectra and (C) ANS fluorescence intensities for proteins (Red, SOD1 aggregates; orange, apo WT SOD1; blue, holo WT SOD1; grey, BSA).; All protein samples were prepared in PBS at a concentration of 0.1 mg/mL. SOD1 aggregates were taken after incubation with TFE for 5 days, and then characterized.

Since the disordered regions and/or monomerization [21], [22], [23] as the result of the metal-free state of SOD1 have a role in initiating protein aggregation, it is well known that apo SOD1 species (non metal bound, usually exists as a monomer state) readily assemble to largely aggregated structures under destabilizing conditions [21], [22], [23], [24], [25], [26], as shown in the description of Figure 2A. Moreover, many recent publications [24], [27] have presented compelling evidence that wild-type (WT) and mutant SOD1 (i.e., especially their apo forms) share an aberrant conformation and a common pathogenic pathway in ALS. In most cases, some of holo mutants largely retain the stability of holo WT SOD1, which is an exceptionally stable form. Related with this, it has been reported that some of the holo mutant SOD1s actually are more stable than apo WT SOD1 [24]. In addition, recent report presenting that the overexpression of WT SOD1 accelerates disease onset of a G85R SOD1 mouse [28], supports the pathogenic mechanism involved with WT SOD1 in ALS. For this reason, we have first studied with aggregates, prepared from apo WT SOD1 as a starting material, to explore the *in vitro* membrane behavior related with SOD1 aggregates. The *in vitro* formation of an SOD1 aggregate was achieved using apo WT SOD1 following a previously reported protocol based on a treatment with 2,2,2-trifluoroethanaol (TFE) [26]. TFE has been extensively used to study the structural variations of other proteins [29], [30]. An inset of Figure 2A shows the general morphology of the as-prepared SOD1 aggregates. The morphological properties of the aggregates prepared *in vitro*, were also characterized by AFM imaging (Figure S1). The sizes and the appearance of amorphous granular structures and rough surfaces, were similar to those reported for ALS patients [31], [32] and ALS transgenic mice [26], [33], [34]. The circular dichroism (CD) spectrum (Figure 2B) of apo WT SOD1 shows the characteristic β-sheet pattern, with a broader peak than that for holo WT SOD1, due to the structural change caused by the absence of Cu and Zn^2+^, which have both catalytic and structural roles in SOD1 [35]. Note that the secondary structure of holo WT SOD1 is known to be mainly comprised of 60% β-sheet and 30% random coil with the remainder being an α-helix [36], [37], compared to bovine serum albumin (BSA, a negative control, mainly composed of α-helixes). In the case of the SOD1 aggregate, the characteristic signature for secondary structures was not found. Since these conformational changes in proteins are generally accompanied by structural turnover and/or changes in surface residues [2], [38], ANS fluorescence was next applied to determine how the surface property of the proteins are altered during the aggregation. A higher ANS fluorescence (5.5-fold enhancement, Figure 2C) was observed for the SOD1 aggregate, compared to its native state (holo WT SOD1). This is caused by the exposure the numerous hydrophobic residues on the protein surface during the aggregation process. Native proteins (holo WT SOD1 and BSA) did not show significant ANS fluorescence enhancement from the base line for a control sample (ANS dye only), while substantial ANS fluorescence enhancement (2.5-fold) for the apo WT SOD1 was observed. Based on our previous report [22], the change in hydrophobicity of a protein significantly affects on the interaction with lipid molecules, which are main components of the cell membrane.

### Monitoring of interactions between the SOD1 proteins and SLB

**Figure 3:**
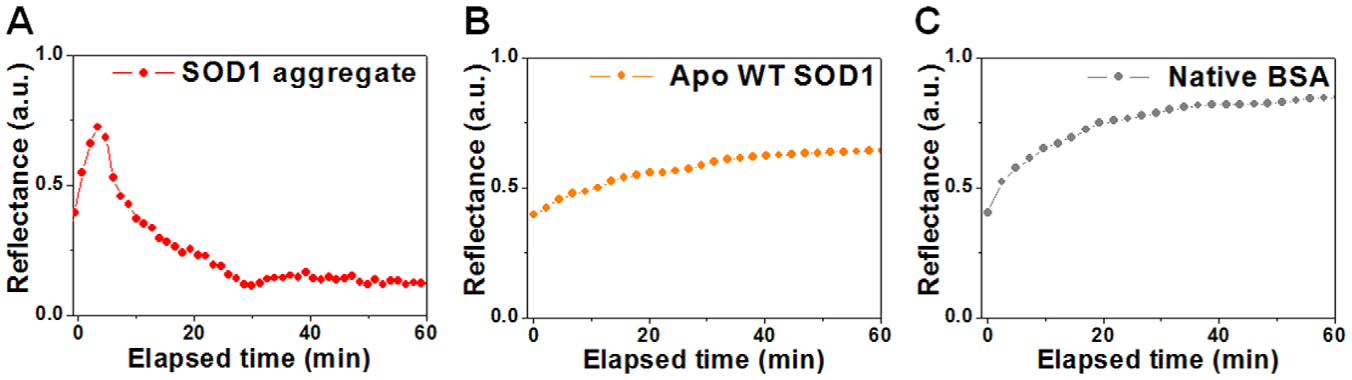
SPR reflectance changes after the interactions between the proteins and the SLBs. (A) SPR reflectance decreases after the interaction between SOD1 aggregates and SLB. (B) SPR reflectance increases after the interaction between apo WT SOD1 and SLB. (C) SPR reflectance increases after the interaction between BSA and SLB. All proteins were prepared in PBS at a concentration of 0.1 mg/ml and were exposed to the lipid membrane for 1 h. The time-resolved reflectance changes derived by the interaction between SLBs and injected proteins were measured using the fixed angle method.

Having shown that SOD1 aggregates have abnormal properties (i.e., structural disorderness and hydrophobicity) compared to their native state, we explored the interactions between the cellular membrane and the SOD1 aggregates, using the methods described in Figure 1B and 1C. We first used a supported lipid bilayer (SLB) prepared from phospholipids (1,2-dipalmitoyl-sn-glycero-3-phosphocholine, DPPC, saturated lipid molecule). An SPR instrument equipped with a reaction cell was used to monitor the interaction between the SLB and the proteins in real-time. A flow cell was mounted onto the SLB/Au/prism assembly so that protein solutions could be easily introduced and flow across the SLB. Interestingly, exposure to the SOD1 aggregate resulted in a decrease in SPR reflectance (Figure 3A and Figure S2A), whereas an increase due to the adsorption to SLB was observed for both apo WT SOD1 and BSA (Figure 3B and 3C). The decrease or increase in SPR reflectance can be attributed to changes in the mass of the SLBs adjacent to the gold film, which is induced by interactions between SLBs and proteins. When the SOD1 aggregates were exposed to the cholesterol contained DPPC SLB, the decrease in SPR reflectance was also observed (Figure S2B).

**Figure 4:**
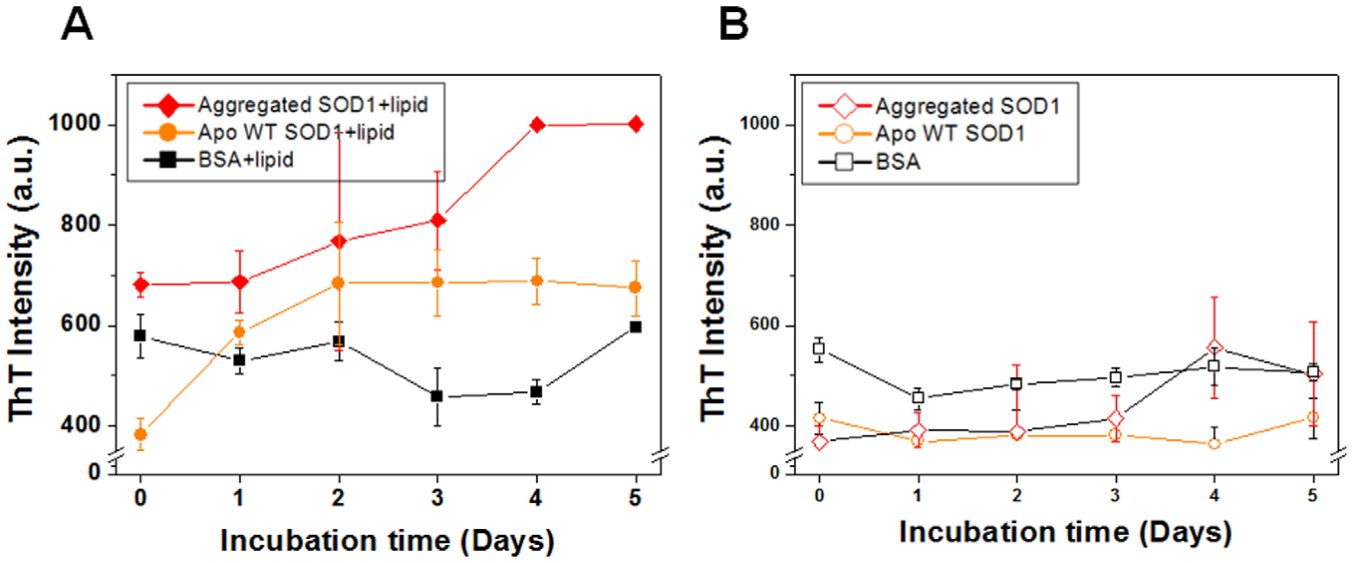
Time-resolved ThT fluorescence measurement for the incubated proteins. (A) With DPPC lipid vesicles. (B) Without DPPC lipid vesicles. To investigate the effect of the interaction of lipid molecules with proteins on aggregation, proteins were incubated with DPPC vesicles in PBS at 37.5°C. As controls, proteins without DPPC vesicles were also incubated under the same conditions. All samples were incubated without agitation. At each time point, an aliquot of each sample was taken and the presence of aggregates was determined using ThT fluorescence assay.

These interactions were also checked by the detection of aggregates induced by the interactions between DPPC vesicles and proteins. After incubating the proteins with DPPC vesicles, they were sampled and then subjected to thioflavin T (ThT) fluorescence measurements. ThT is a fluorescence probe used to detect the presence of aggregates and the amount of aggregated proteins [26], compared to the initial state of each sample. Figure 4A shows the results for the time-resolved analysis for the further SOD1 aggregation induced by the interaction between proteins and lipid vesicles. As controls, we also analyzed the proteins incubated without lipid vesicles (Figure 4B). No significant changes were observed in the control cases. While, SOD1 aggregates, when incubated with lipid vesicles, demonstrated a higher tendency to undergo aggregation than the other samples depending on the incubation time. In the case of apo WT SOD1, the fluorescence intensity also increased to a considerable extent. This indicates that lipid-SOD1 complex [22] can be formed on a SLB. Accordingly, the decrease (Figure 3A) or increase (Figure 3B and 3C) in SPR reflectance can be attributed to the detachment of lipid molecules on the SLB and binding to the SLB, respectively, which are induced by interactions between SLBs and proteins. To demonstrate the relevance of the observed strong binding affinity of the SOD1 aggregates to the saturated DPPC lipid molecules, we also examined the binding propensity with other lipid vesicles composing different compositions (i.e., unsaturated lipid molecules and cholesterol molecules). In the case of 1-palmitoyl-2-oleoyl-phosphatidylcholine (POPC, unsaturated lipid molecule) lipid vesicles (Figure S3), the insertion of cholesterol molecules to the vesicles induced the significant increase of the fluorescent intensity compared to the cases of POPC lipid only and cholesterol only. The association propensity was also different among different mole fraction between lipid molecules and cholesterol molecules (i.e., 1∶0, 1∶1, 2∶1, and 0∶1). This result indicates that the interactions between lipid vesicles and SOD1 aggregates can behave differently dependent on the saturation of lipid molecule chain and the composition of vesicles.

**Figure 5:**
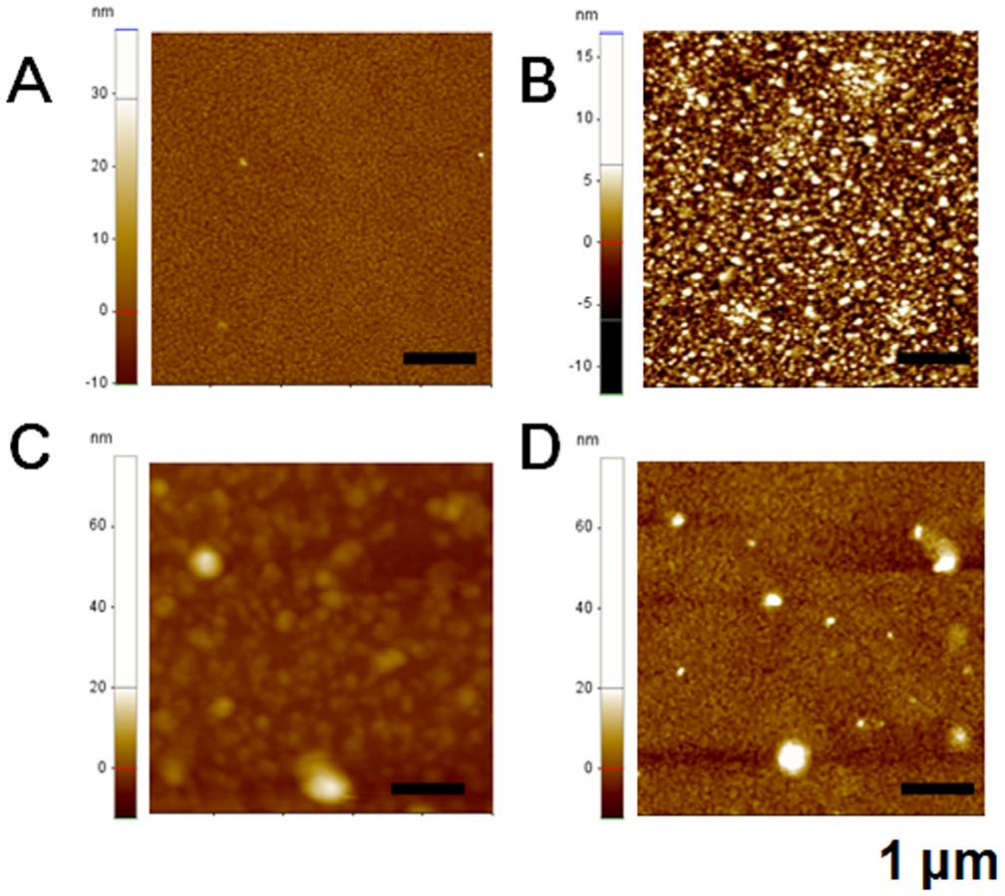
AFM images of resulting SLB surfaces. (A) Original SLB layer. (B), (C), and (D) SLB layers after the interaction with SOD1 aggregates, apo SOD1, and BSA, respectively. Bars, 1 µm. All images were obtained after the SPR measurement.

AFM images of the SLB surface were also obtained to visualize and cross-check the results of the SPR measurements (Figure 3) for changes in SLB and the ThT assay (Figure 4) for resulting aggregates induced by molecular interactions between proteins and lipid vesicles. Figure 5A shows an AFM image of the original SLB surface formed on a gold substrate, which resembled the morphology of the gold substrate (Figure S4). It should be noted that the roughness of a gold surface was below 2 nm and that of undamaged SLB surface was also below 2 nm. After the exposure of SLB to SOD1 aggregates, numerous defects within the SLB were observed (Figure 5B), compared with the SLB before exposure (Figure 5A). In the case of apo WT SOD1, an AFM image (Figure 5C) shows a considerable increase in the number of clusters on the original smooth SLB surface, which is due to the formation of a lipid-protein complex, as supported by the ThT fluorescence measurement of the interaction between apo WT SOD1 and lipid molecules (Figure 4A). Conversely, native BSA was bound to the SLB and its integrity was maintained (Figure 5D), which is attributable to its non-specific binding property and poor aggregation propensity after incubation with lipid molecules, as confirmed by the ThT assay (Figure 4A). From the results, we conclude that the SOD1 variants with disordered regions, as the result of demetallation (apo SOD1) and further aggregation (SOD1 aggregate), can cause the loss of SLB integrity, as the result of further aggregation on a SLB and/or the detachment of parts of the SLB (i.e., defects), respectively.

### Identification of defects within the SLB by interaction with the SOD1 aggregate

**Figure 6:**
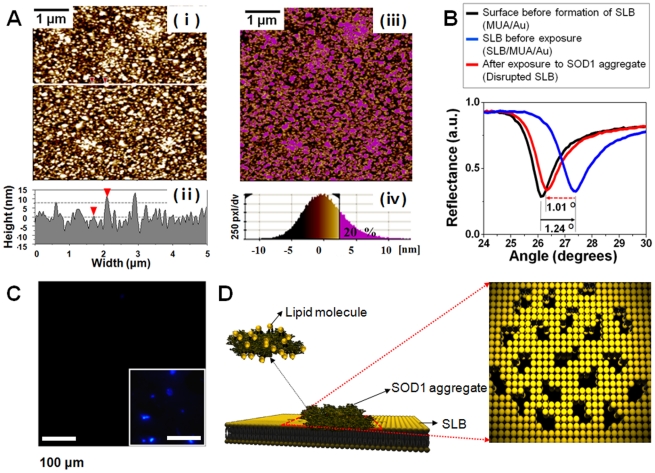
The formation of defects within the SLB by the interaction between the SOD1 aggregate and the SLB. (A) (π) and (θ) AFM image and height profile of the resulting SLB surface after the exposure to the SOD1 aggregate. (ρ) and (σ) AFM image colored for remaining lipid layers (shown in pink) and height distribution of entire surface. Remaining lipid layers occupy approximately 20% of the entire surface. (B) SPR contour plots at each event. Each plot indicates before formation of SLB (black), after formation of SLB (blue), and after interaction between SLB and SOD1 aggregates (red), respectively. (C) ThT fluorescence image of the resulting SLB surface. (An inset shows clear blue ThT fluorescence for a SOD1 aggregate solution.). Bars, 100 µm. (D) Description of the formation of defects within a SLB by the SOD1 aggregate.

We next performed an in-depth characterization of SLBs that contained numerous defects. Figure 6A shows a line profile of the resulting SLB (Figure 5B), caused by contact with SOD1 aggregates. The data show a remarkable height difference between the bright domain and the dark domain of less than 8 nm (indicated by red arrow-heads). Considering that the length of a DPPC molecule is 3.4 nm [39], the bright and dark domains correspond to remaining lipid layers and lipid-detached regions, respectively. As shown in the right image of Figure 6A, the remaining lipid layers (shown in pink) occupy approximately 20% of the entire surface. This is in good agreement with the SPR result, in which the loss of surface coverage by SLB was calculated to be 80% (Figure 6B, Since SPR angular shift is typically proportional to the surface coverage [40], we calculated the change in coverage from the collected SPR angles for the SLB before and after its contact with the SOD1 aggregates, which were 1.24° and 1.01°, respectively. We also observed the similar decrease in the surface coverage of cholesterol contained DPPC SLB after exposure to the SOD1 aggregates (Figure S2B, S2C). This result clearly indicates that the changes in lipid membrane integrity by interacting with the SOD1 aggregates are common behaviors irrespective of the existence of cholesterol molecules. In addition, we characterized the size of the defects based on the AFM characterization for the SLB after its exposure to the SOD1 aggregates. As shown in Figure S5, we can obtain the statistic data from the surface topography (Figure 6A) of the resulting lipid membrane. Figure S5B shows the grains indicating the dark areas (i.e., lipid detached areas) of the Figure S5A. Histograms (Figure S5C, S5D) show the statistic distribution of the length and area of the numbered areas in the Figure S5B, respectively. Result shows that the majority of the defects have several hundreds of lengths and areas of below 0.1 µm^2^.

ThT fluorescence imaging (Figure 6C) was applied to SLB, to determine whether or not the aggregate remains on the surface of the SLB after the interaction. The inset shows a clear blue ThT fluorescence for the SOD1 aggregate solution exposed to the SLB layer, however, visible blue spots were not found over most of the field of view for the resulting SLB, indicating that no aggregate remains on the surface of the SLB. This further indicates that, after the interaction between an aggregate and SLB, the aggregate assembled with lipid molecules is detached from the SLB, thus creating numerous defects within the SLB, as shown in the description of Figure 6D.

### Identification of unregulated membrane permeability in HEK 293 cell membrane patches by SOD1 aggregates

It should be noted that the formation of defects within a membrane by proteins is extremely important because the perturbation in mass transport (i.e., membrane permeability by defect) may play crucial roles in determining membrane-mediated cytotoxicity [18], [41]. Our finding of the formation of defects is also supported by recent reports of membrane disruption by Amyloid-β fibrils [18], [20] and amyloidogenic human amylin peptide [19], [41]. Although the changes in membrane integrity could constitute a viable candidate for membrane permeabilization, membrane permeabilization induced by protein aggregates has been mainly investigated so far by indirect demonstrations, such as monitoring the kinetics of a fluorescent probe released from vesicles [18], [19] and molecular simulations with proteins and vesicles [20]. By monitoring the fluorescent dye released from the DPPC lipid vesicles, we pre-observed the permeability of lipid membrane (Figure S6). For a more practical demonstration, we examined what occurs in a real cell membrane system, as the result of changes in membrane integrity by the formation of defects and the binding to cell membranes observed in our model system, SLB. For a direct demonstration of membrane permeability with a cellular membrane, we investigated membrane permeability by measuring current changes in membrane patches detached from HEK 293 cells upon exposure to different SOD1 variants.

**Figure 7:**
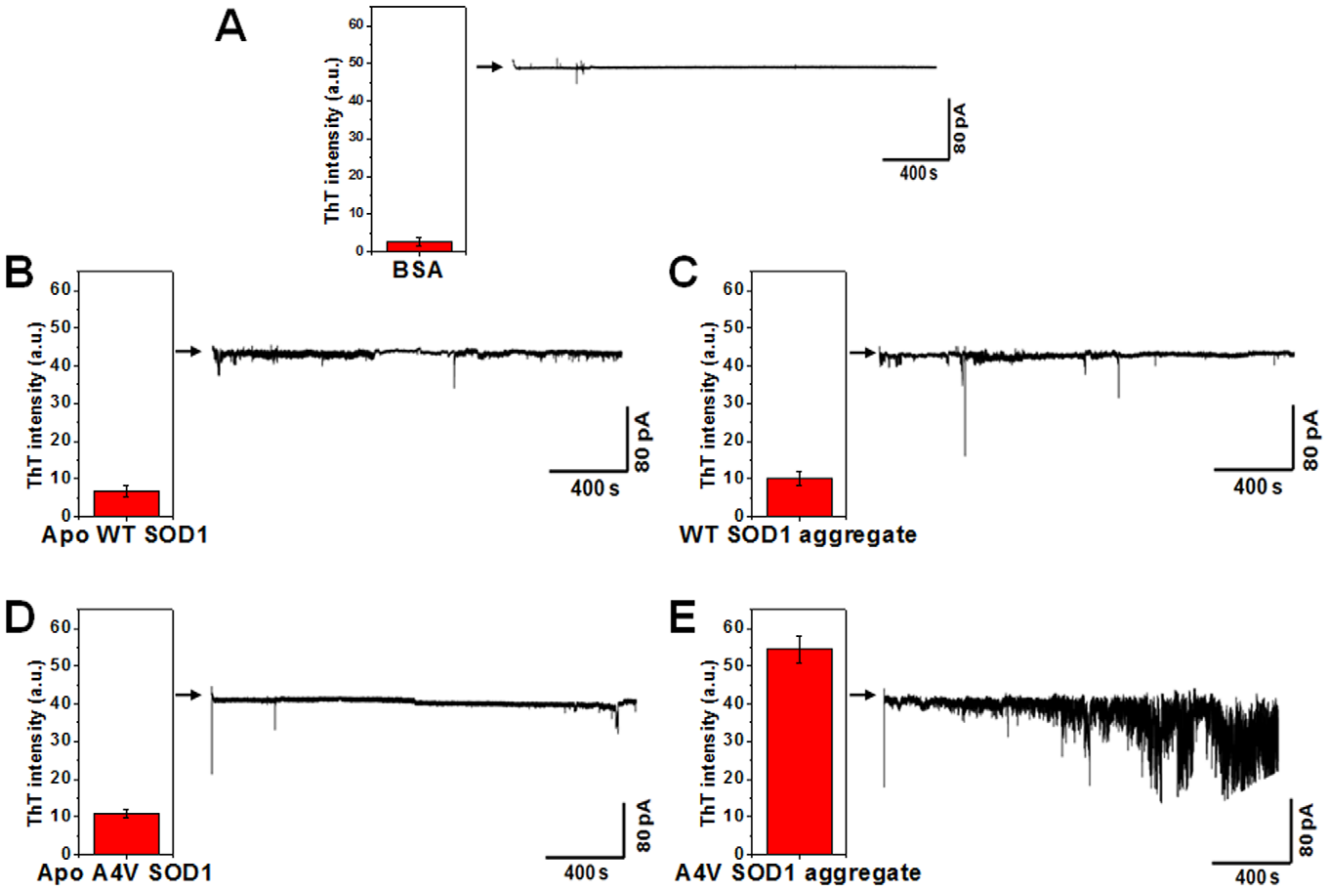
The unregulated membrane permeability in HEK 293 cell membrane patches by the SOD1 aggregates. The ionic permeability of HEK 293 cell membranes in response to the perfusion of proteins was measured at 10 kHz in inside-out configuration (Fig. 1C) of a patch clamp. The bath (approximately 0.15 ml) was superfused at 1 ml/min and voltage clamp experiments were performed at room temperature (22–25°C). (A–E) Electrophysiological recordings for (A) BSA, as a control, (B) Apo WT SOD1, (C) WT SOD1 aggregate, (D) Apo A4V SOD1, and (E) A4V SOD1 aggregate. Bar graphs represent the ThT fluorescence intensity of each sample solution obtained before perfusing.

In this test, to collect more biologically relevant information (i.e. mutation effect) through the use of a real cell membrane patch, A4V SOD1 as well as WT SOD1 was chosen for the following reasons; (1) We suspected that membrane permeability might be strongly dependent on the extent of aggregation, and it is well known that the kinetics of aggregation for A4V are faster than that of WT SOD1 [42], [43]. (2) The A4V mutation is one of several ALS-linked SOD1 mutations. As shown in Figure S7A, S7B, the A4V SOD1 aggregate forms a much greater extent than that of WT SOD1 under the same conditions due to the difference in aggregation kinetics, which is consistent with previous reports [21], [42], [43]. However, they share common properties of aggregates in terms of surface property and morphology, as judged from the ANS fluorescence enhancement (Figure S7C) and the electron micrographs (Figure S7D). For as-prepared SOD1 variants, the ionic permeability of HEK 293 cell membranes (Figure 7) was measured using the inside-out configuration (a description of Figure 1C) of a patch clamp. A large increase in conductance was observed on perfusing the A4V SOD1 aggregate into the experimental bath (Figure 7E). In stark contrast, no significant change in conductance was observed in the control with BSA (Figure 7A), which can be attributed to the conservation of the integrity of membrane after being in contact with BSA, as observed in the SLB (Figure 5D). For holo SOD1, no obvious change was also observed (data not shown). However, WT apo SOD1 and A4V apo SOD1 (Figure 7B and 7D) slightly changed conductance, which might be due to membrane binding and further aggregation with lipid molecules induced by their structural disorderness, based on the results observed for SLB (Figure 5C). The WT SOD1 aggregate often increased the membrane conductance (Figure 7C) but with far less frequency than the A4V SOD1 aggregate (Figure 7E). Since such changes in conductance are closely related to unregulated membrane permeability, it appears likely that the SOD1 aggregates induce the formation of numerous defects within the cellular membrane, thus perturb membrane permeability. As expected, the changes in extent of the conductance were correlated with the amount of aggregates present, as confirmed by ThT fluorescence intensity data (each inset of Figure 7). Taken together, different conformations at the metal-free state did not induce significant difference in conductance changes (Figure 7B and 7D). However, when they form aggregates, remarkable difference was observed according to its original conformation (Figure 7C and 7E). On the basis of these results, we can address that membrane permeabilization is more strongly related with the quantity of resulting aggregates rather than original conformations at their native and/or monomer states. This can be supported by the recent reports showing that WT and mutant SOD1 share an aberrant conformation and a common pathogenic pathway in ALS [24], [27].

## Discussion

We note that interactions between protein aggregates and cell membranes may play a crucial role in determining the membrane-associated cytotoxicity of aggregated proteins, because many protein conformation diseases (i.e., Alzheimer's disease, Parkinson's disease, prion disease, and ALS) are known to be related to membrane system [7], [18], [20], [44], [45], [46]. In this study, we for the first time demonstrate the cytotoxic actions of SOD1 aggregates associated with membranes by showing their abnormality in terms of structural and surface properties, and by investigating changes in the membrane integrity and permeability. Based on our observations, it appears that the aggregates with abundant hydrophobic parts, induced by conformational changes, can be readily inserted to the inner space of the membrane and assembled with the hydrophobic tails of lipid molecules. This is well supported by our previous work [22] showing that the change in hydrophobicity of a protein significantly affects on the interaction with lipid molecules and finally induces the formation of cytotoxic aggregation. Although the binding interactions between the SOD1 aggregates and lipid membranes are affected by various factors including lipid structures and lipid compositions, the changes in the lipid membrane integrity by interacting with SOD1 aggregates were found to be common behaviors. As a result, defects within a membrane are formed by the detachment of lipid molecules that are co-assembled with SOD1 aggregates. This *in vitro* observation was also directly examined by using real cell membrane patches with more complicated compositions and structures than artificial SLBs. Basically, the extent of changes in permeability through membrane patches depended on the amount of aggregates.

In conclusion, our findings suggest that a plausible explanation for the changes in membrane integrity and permeability is the structural disorderness and surface hydrophobicity of SOD1 aggregates, which are induced by conformational changes. That is, the abnormal interaction between a membrane and SOD1 aggregates induces defects in membrane integrity, and perturbs membrane permeability. We expect that this finding will advance our understanding of the pathway by performing further through investigations of the gain of toxic function of SOD1 variants and offer diagnostics and therapeutics associated with ALS. To more clearly address our observations, more investigations on other structurally disordered proteins (i.e., Amyloid-β fibrils) as well as other mutant SOD1s are currently underway by employing *in vitro* observation methods used here. We believe that these fundamental studies of biomolecular interactions can be a model system and provide clues for understanding the intra- and extracellular interactions between real cell membranes and proteins.

## Materials and Methods

### Materials

1,2-dipalmitoyl-sn-glycero-3-phosphocholine (DPPC), 1-palmitoyl-2-oleoyl-phosphatidylcholine (POPC), bovine serum albumin (BSA), and thioflavin T (ThT) were purchased from Sigma. 2,2,2-trifluoroethanaol (TFE) was purchased from Aldrich. 11-mercaptoundecanoic acid (MUA) and 8-anilino-1-naphthalene-sulfonic acid (ANS) were obtained from Sigma-Aldrich. Standard 10× phosphate buffered saline (100 mM PBS), which contained the salts; NaCl 80.0 g (1.37 M), KCl 2.0 g (27 mM), Na_2_HPO_4_ 14.4 g (100 mM), KH_2_PO_4_ 2.4 g (17.6 mM), was diluted to the 25 mM PBS for our all experiments. All chemicals were used as received without further purification.

### *In vitro* preparation of SOD1 aggregate

The purification of SOD1 proteins was achieved by following a previously reported purification protocol [16], [22]. To prepare SOD1 aggregate, the purified apo SOD1 samples were diluted with an acidic phosphate buffer saline (PBS) solution (pH 5.4) at a concentration of 0.1 mg/ml [26]. SOD1 aggregates were prepared by treatment with a solution containing 20% (v/v) TFE. To monitor aggregate formation, ThT fluorescence of samples was measured by fluorescence spectroscopy (LS 55, PerkinElmer). ThT is known to associate rapidly with aggregated fibrils, giving rise to a new excitation maximum at around 450 nm and an enhanced emission at around 482 nm. 150 µL of each sample was mixed with 15 mL of 10 µM ThT in PBS, and fluorescence emission intensity was immediately and repeatedly recorded at least five times to obtain a reasonable values. To further characterize the morphology of the SOD1 aggregates, transmission electron microscopy (TEM) was used. Samples (150 µL aliquots) were incubated on 400-mesh carbon-coated formvar copper grids overnight. The grids were air-dried and stained with 1% (w/v) uranyl acetate. Specimens were viewed with an energy-filtering TEM (LIBRA, Carl Zeiss) at an accelerating voltage of 120 kV.

### Circular dichroism (CD) spectroscopy

The secondary structure of proteins was determined by CD analysis (Jasco J-715 spectropolarimeter). A 0.1-cm quartz cell was used for the measurements and the CD spectra were recorded from 190 to 250 nm. All CD measurements were carried out using the following parameters: 1 nm bandwidth, 50 nm/min run speed, 1 nm step size, 8.3 s response time, and an average of three runs.

### ANS fluorescence spectroscopy

For the measurement of surface hydrophobicity, the fluorescence property of the extrinsic fluorophore ANS (final concentration 20 µM, excitation at 360 nm, bandwidth 5 nm) was examined by 10 min of incubation at 37°C with proteins in the buffer via fluorescence spectroscopy.

### Preparation of lipid vesicles and SLBs

The vesicles were prepared by probe sonication. A 40 mg portion of lipids was dissolved and agitated in 4 mL chloroform in a glass vial. For the preparation of cholesterol contained lipid vesicles, cholesterol molecules were added to the lipid solution by the desired molar ratios. Freeze-and-thaw cycles by liquid nitrogen for three times. The organic solvent was evaporated with a nitrogen stream to form a thin lipid layer on the inner wall of the vial. Dried lipid films were resuspended in PBS at a concentration of 0.1 mg/mL, and the suspension was sonicated at low amplitude in an ice bath for 30 min. Sonication for 5–10 min was repeated to avoid too much increase in the suspension temperature. The resulting vesicle solution was then stored at 4°C before use. For the preparation of SLBs, Au substrate was modified by pretreatment of 5 mM MUA ethanolic solution for overnight and was rinsed with pure ethanol to remove the excess molecules. The resulting hydrophilic substrate was incubated under 100 µL of the vesicle suspension for 2 h at 45°C. After incubation, the excess vesicles were removed by rinsing with PBS.

### *In situ* SPR/SLB assays and AFM imaging

For the real-time analysis of interaction between SLBs and proteins, commercial SPR instrumentation (K-MAC) equipped with reaction cell was used [16]. Specimens of SOD1 aggregates, apo SOD1, and native BSA were separately dispersed in PBS at a concentration of 0.1 mg/ml. Each specimen was exposed to the lipid membrane for 1 h. The time-resolved reflectance changes derived by the interaction between SLBs and injected samples were measured using the fixed angle method. The change in surface coverage of the remained SLB was calculated from the collected SPR angles, since SPR angular shift is typically proportional to the surface coverage [40]. After the incubation with each protein solution, the resulting morphologies of SLBs were also characterized by AFM imaging. AFM images were acquired in non-contact mode using an atomic force microscope (XE-100, Park systems). Uncoated silicon cantilevers with typical tip curvature radius of about 10 nm were used.

### Time-resolved analysis of lipid molecule-induced aggregation

To investigate the effect of the interaction of lipid molecules with proteins on aggregation, proteins were incubated with DPPC vesicles in PBS at 37.5°C. As controls, proteins without DPPC vesicles were also incubated under the same conditions. All samples were incubated without agitation. At each time point, an aliquot of each sample was taken and the presence of aggregates was determined using ThT fluorescence assay.

### Cell culture

Human embryonic kidney (HEK 293) cell line was obtained from American Type Culture Collection (Manassas, VA, USA). Prior to the experiment, HEK 293 cells were grown in Dulvecco's Modified Eagle's Medium (DMEM) (Invitrogen) supplemented with 10% (v/v) heat-inactivated fetal bovine serum (Hyclone, Logan, UT), (Sigma), and 1% penicillin/streptomycin (Invitrogen). All cells were incubated in 20% O_2_, 10% CO_2_ 37°C.

### Electrophysiological recordings

For electrophysiological recordings, the cells were transferred into a bath mounted on the stage of an inverted microscope (IX-70, Olympus). The bath (approximately 0.15 ml) was superfused at 1 ml/min and voltage clamp experiments were performed at room temperature (22–25°C). Patch pipettes with a free-tip resistance of about 2.5 MΩ were connected to the head stage of a patch-clamp amplifier (Axopatch-1D, Axon Instruments). pCLAMP software v.9.2 and Digidata-1322A (both from Axon Instruments) were used for the acquisition of data and the application of command pulses. Single channel activities were recorded at 10 kHz in inside-out configurations. The pipette solution contained (in mM) 145 KCl, 1 EGTA, and 10 HEPES with a pH of 7.4 (titrated with KOH). The bath solution contained (in mM) 145 KCl, 1 EGTA, and 10 HEPES with a pH of 7.2 (titrated with KOH). The voltage and current data were low-pass filtered at 1 kHz. Current traces were stored and analyzed using Clampfit v.9.2 and Origin v. 7.0 (Microcal Inc.).

## Supporting Information

Figure S1 **Morphology of the prepared SOD1 aggregates.** (A) AFM image of the SOD1 aggregates formed after 5 days incubation. Bar, 5 µm. (B) Three-dimensional image of (A) showing amorphous granular structures and rough surfaces. (C) Thickness profile of a red line in (B).
(TIF)

Figure S2 **SPR contour plots for SLBs disruption process according to the regular time-interval exposure of SOD1 aggregates.** (A) Interaction between SOD1 aggregates and DPPC lipid layer (B) Interaction between SOD1 aggregates and DPPC lipid layer with cholesterol domains. (C) Plots for the coverage of SLBs remained according to the interaction time with SOD1 aggregates.
(TIF)

Figure S3 **The interactions between the lipid vesicles and SOD1 aggregates for four types of lipid vesicles with different compositions.**
(TIF)

Figure S4 **Formation of a SLB on a Au substrate.** (A) SPR contour plots before and after formation of SLB on the hydrophilic Au surface. (B) AFM images of a hydrophilic Au surface modified with MUA (left) and a SLB surface formed after vesicle fusion (right). Bars, 500 nm.
(TIF)

Figure S5 **The size and distribution of the defects within lipid membrane based on the AFM characterization.** (A) Surface topography of the resulting lipid membrane after interaction with the SOD1 aggregates. (B) Image assigning the grains where indicate the dark areas (i.e., lipid detached areas) of the Figure S5A. (C) and (D) Statistic distribution of the length and area of the numbered areas in the Figure S5B.; The majority of the defects have several hundreds of lengths and areas of below 0.1 µm^2^.
(TIF)

Figure S6 **Dye leakage assay from the lipid vesicles induced by interactions between the lipid vesicles and SOD1 aggregates.**
(TIF)

Figure S7 **The A4V and WT SOD1 aggregates used in this study share a common morphology, but the kinetics of their aggregation is dramatically different.** (A) ThT florescent intensity of the prepared SOD1 aggregates under same conditions (i.e., same protein concentration and destabilizing condition). (B) Visualization of the aggregates by formation of the precipitation, which are induced by gold nanoparticle binding to the SOD1 aggregates; The A4V aggregate is much more formed that of WT SOD1 under the same conditions (Note that the aggregation kinetic of A4V is faster than that of WT.). (C) and (D) ANS fluorescence enhancement and morphologies of prepared aggregates show the similarity between WT (left) and A4V (right).
(TIF)
