# Supplementary figures and images for: Direct Observation of Defects and Increased Ion Permeability of a Membrane Induced by Structurally Disordered Cu/Zn-Superoxide Dismutase Aggregates
Source: PLoS One. 2011 Dec 28;6(12):e28982. doi: 10.1371/journal.pone.0028982 (PMC3247219; doi:10.1371/journal.pone.0028982)

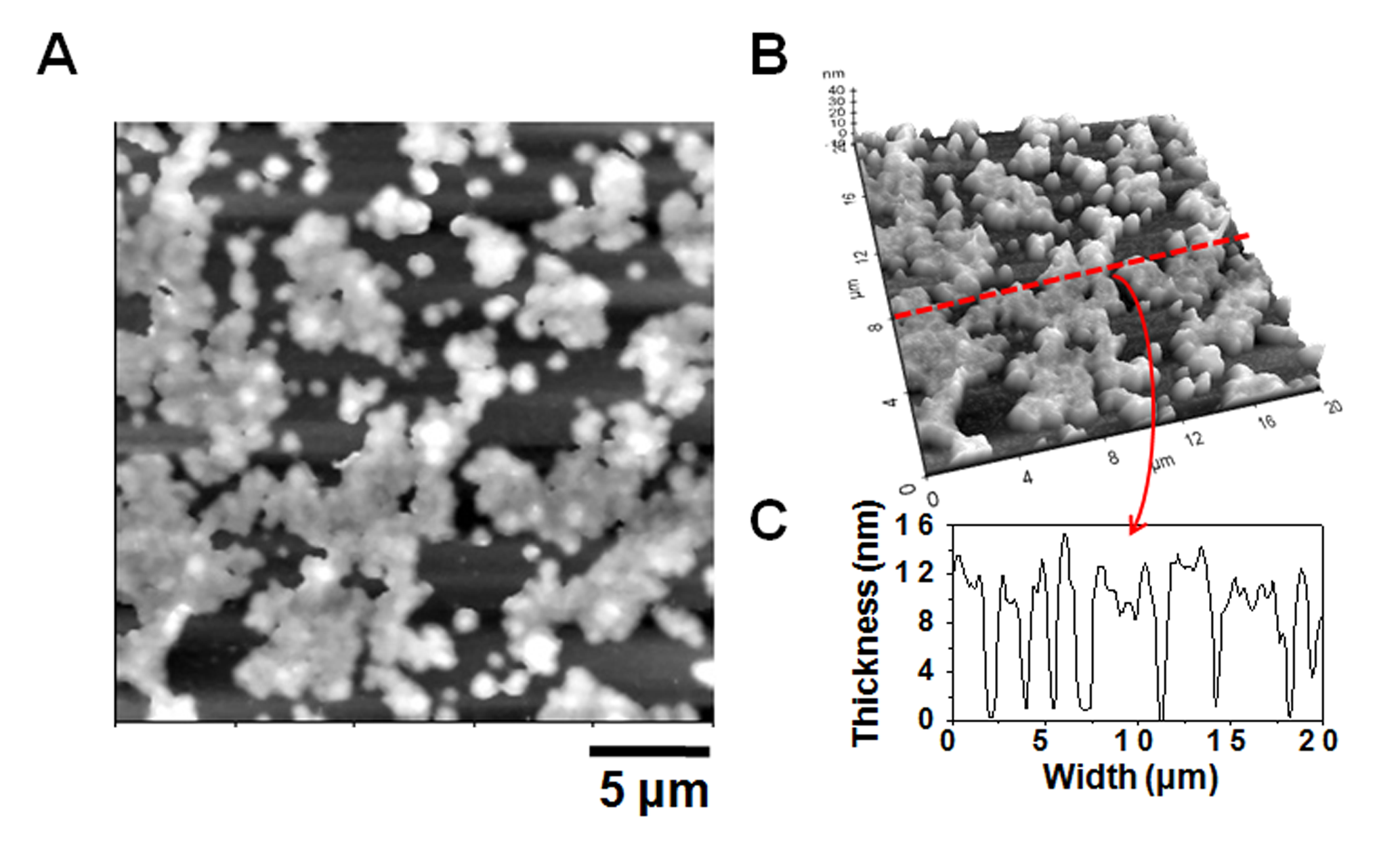

Supplement: Figure S1 — Morphology of the prepared SOD1 aggregates. (A) AFM image of the SOD1 aggregates formed after 5 days incubation. Bar, 5 µm. (B) Three-dimensional image of (A) showing amorphous granular structures and rough surfaces. (C) Thickness profile of a red line in (B). (TIF) [file pone.0028982.s001.tif]

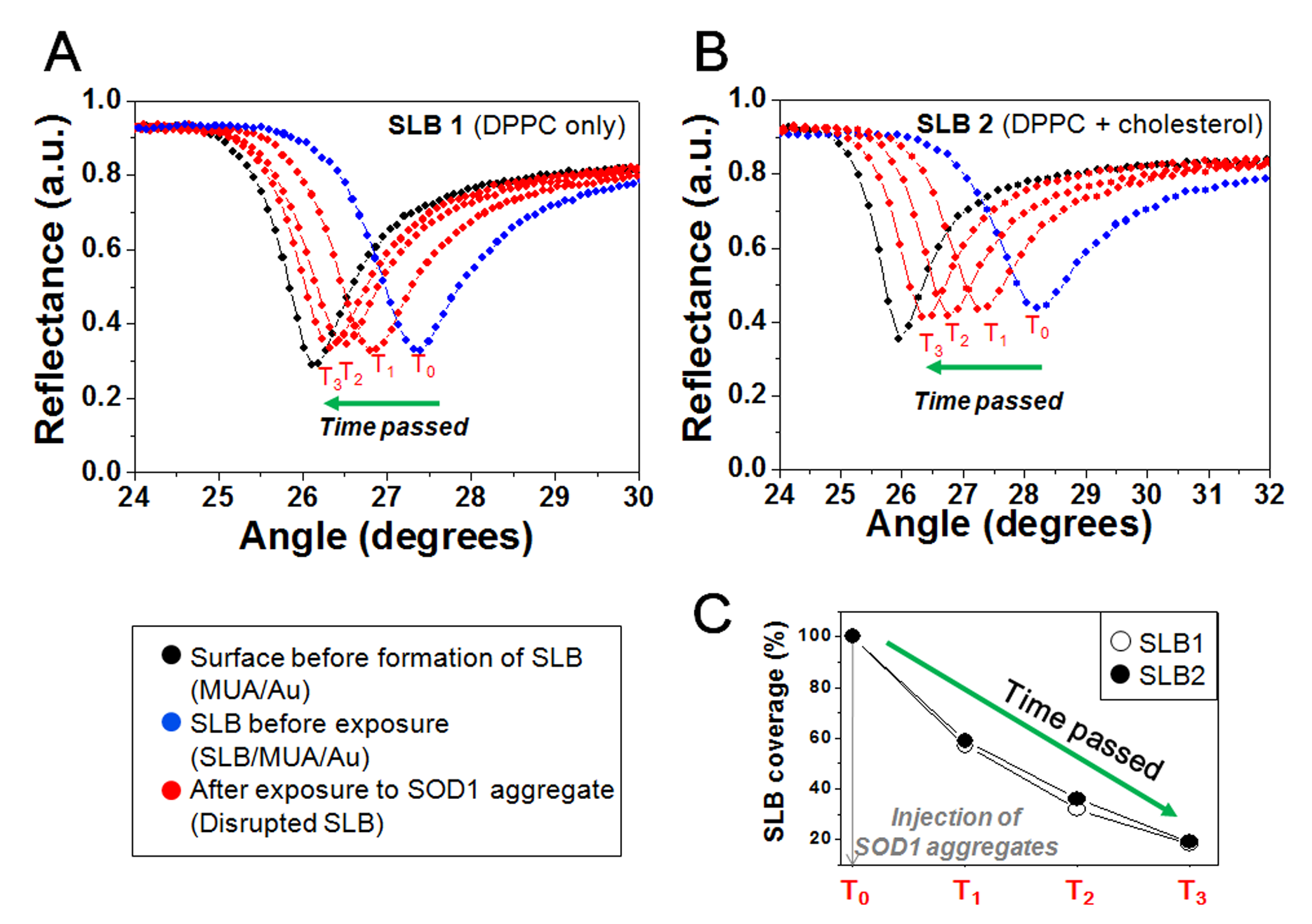

Supplement: Figure S2 — SPR contour plots for SLBs disruption process according to the regular time-interval exposure of SOD1 aggregates. (A) Interaction between SOD1 aggregates and DPPC lipid layer (B) Interaction between SOD1 aggregates and DPPC lipid layer with cholesterol domains. (C) Plots for the coverage of SLBs remained according to the interaction time with SOD1 aggregates. (TIF) [file pone.0028982.s002.tif]

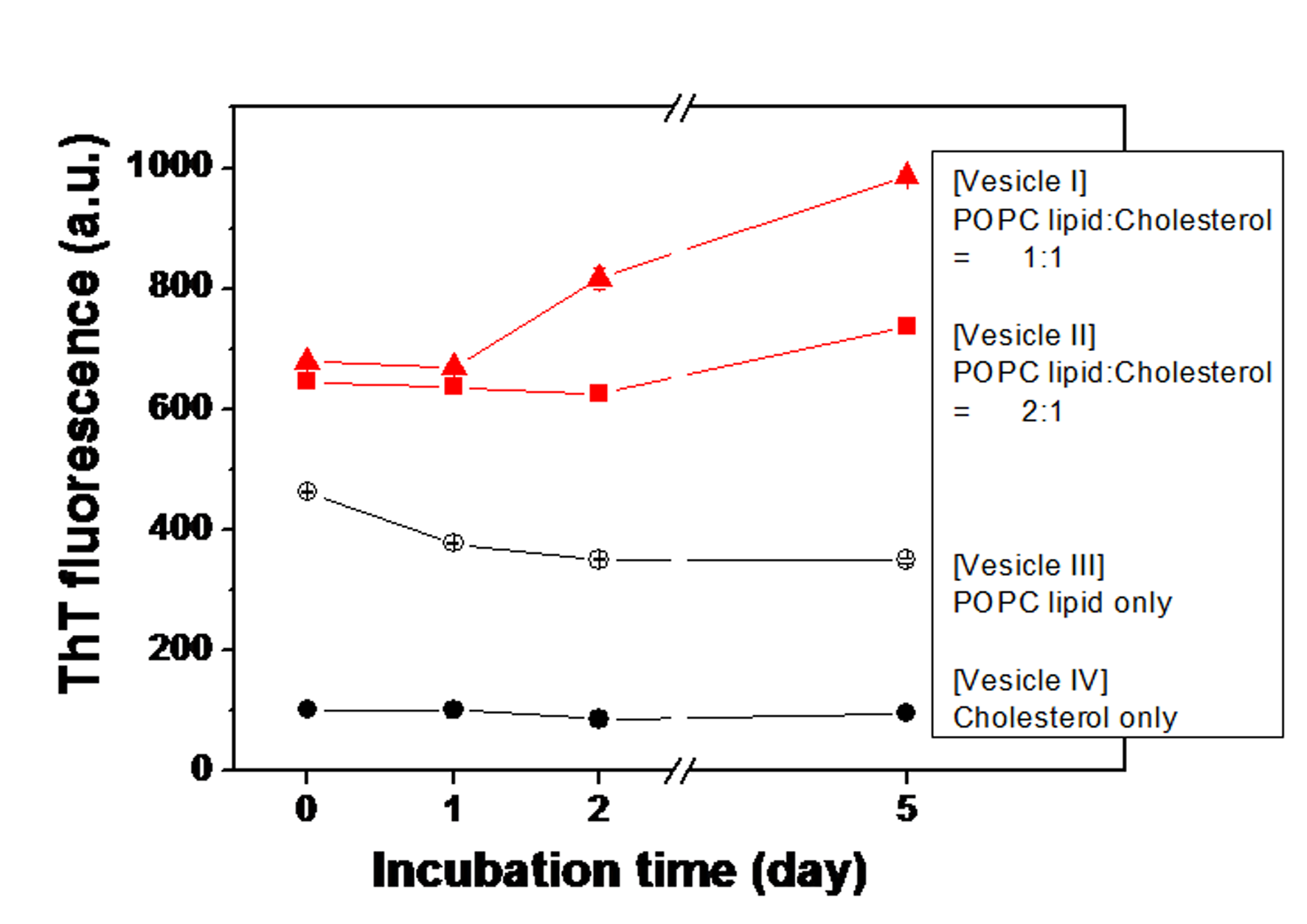

Supplement: Figure S3 — The interactions between the lipid vesicles and SOD1 aggregates for four types of lipid vesicles with different compositions. (TIF) [file pone.0028982.s003.tif]

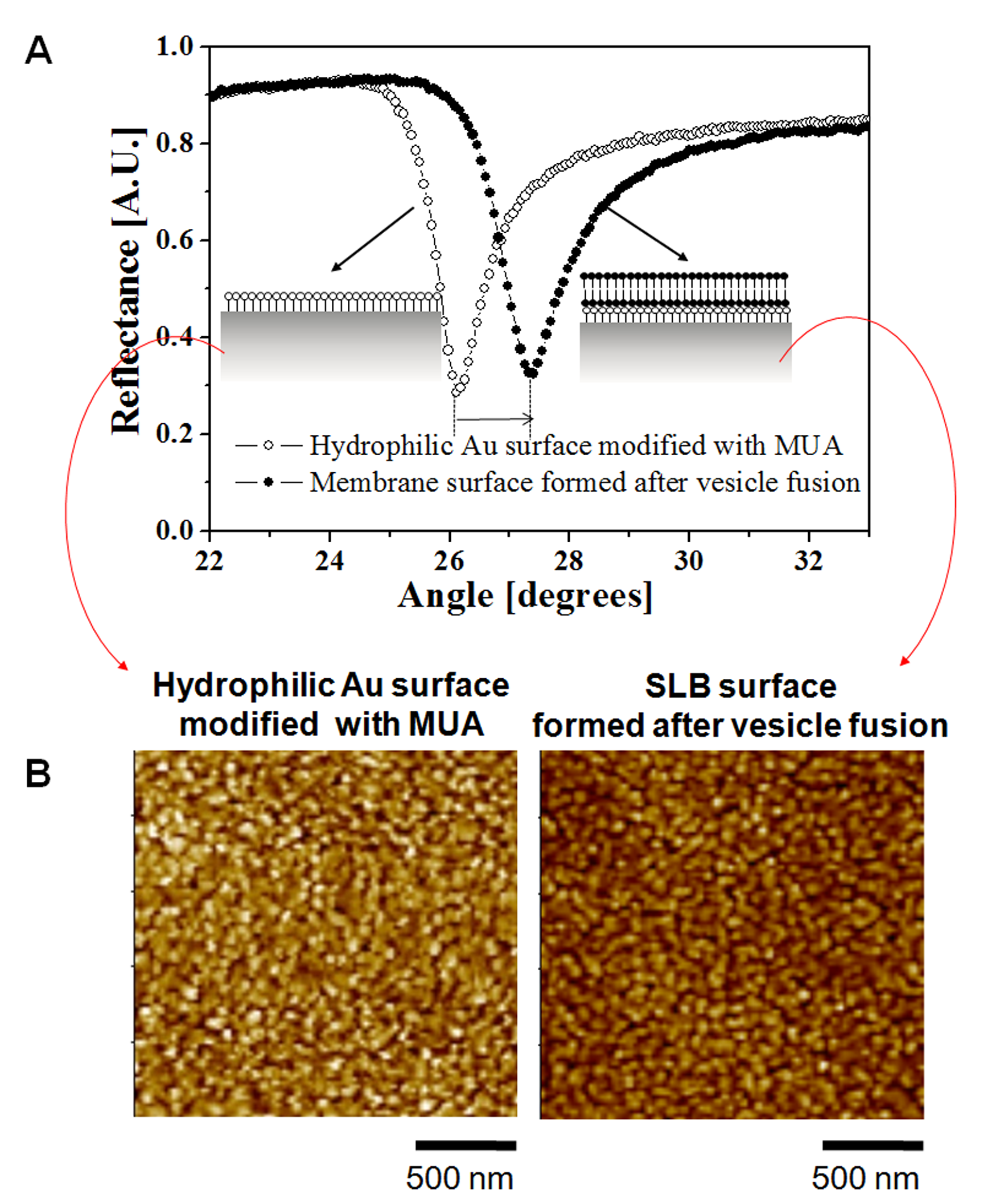

Supplement: Figure S4 — Formation of a SLB on a Au substrate. (A) SPR contour plots before and after formation of SLB on the hydrophilic Au surface. (B) AFM images of a hydrophilic Au surface modified with MUA (left) and a SLB surface formed after vesicle fusion (right). Bars, 500 nm. (TIF) [file pone.0028982.s004.tif]

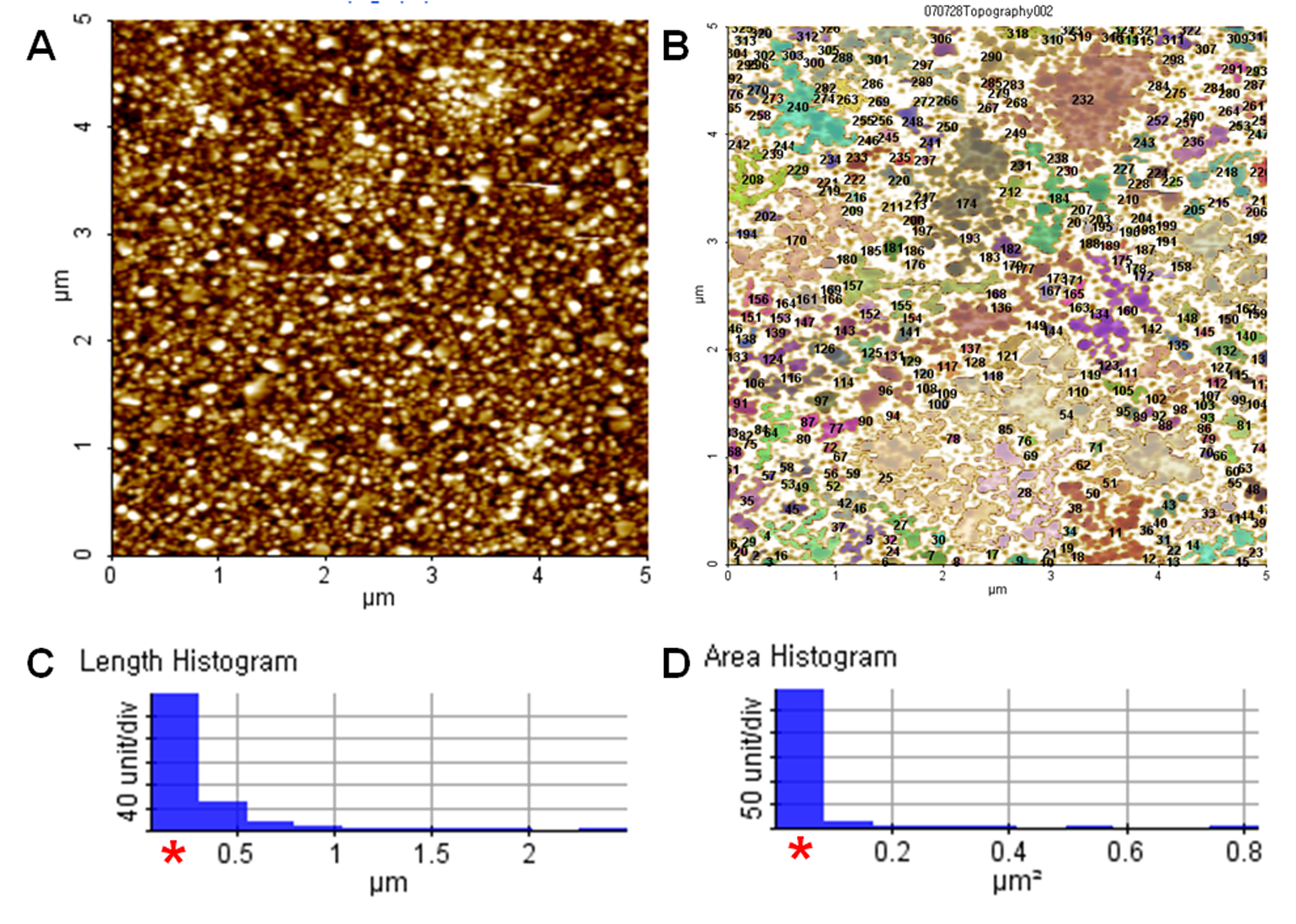

Supplement: Figure S5 — The size and distribution of the defects within lipid membrane based on the AFM characterization. (A) Surface topography of the resulting lipid membrane after interaction with the SOD1 aggregates. (B) Image assigning the grains where indicate the dark areas (i.e., lipid detached areas) of the Figure S5A. (C) and (D) Statistic distribution of the length and area of the numbered areas in the Figure S5B.; The majority of the defects have several hundreds of lengths and areas of below 0.1 µm2. (TIF) [file pone.0028982.s005.tif]

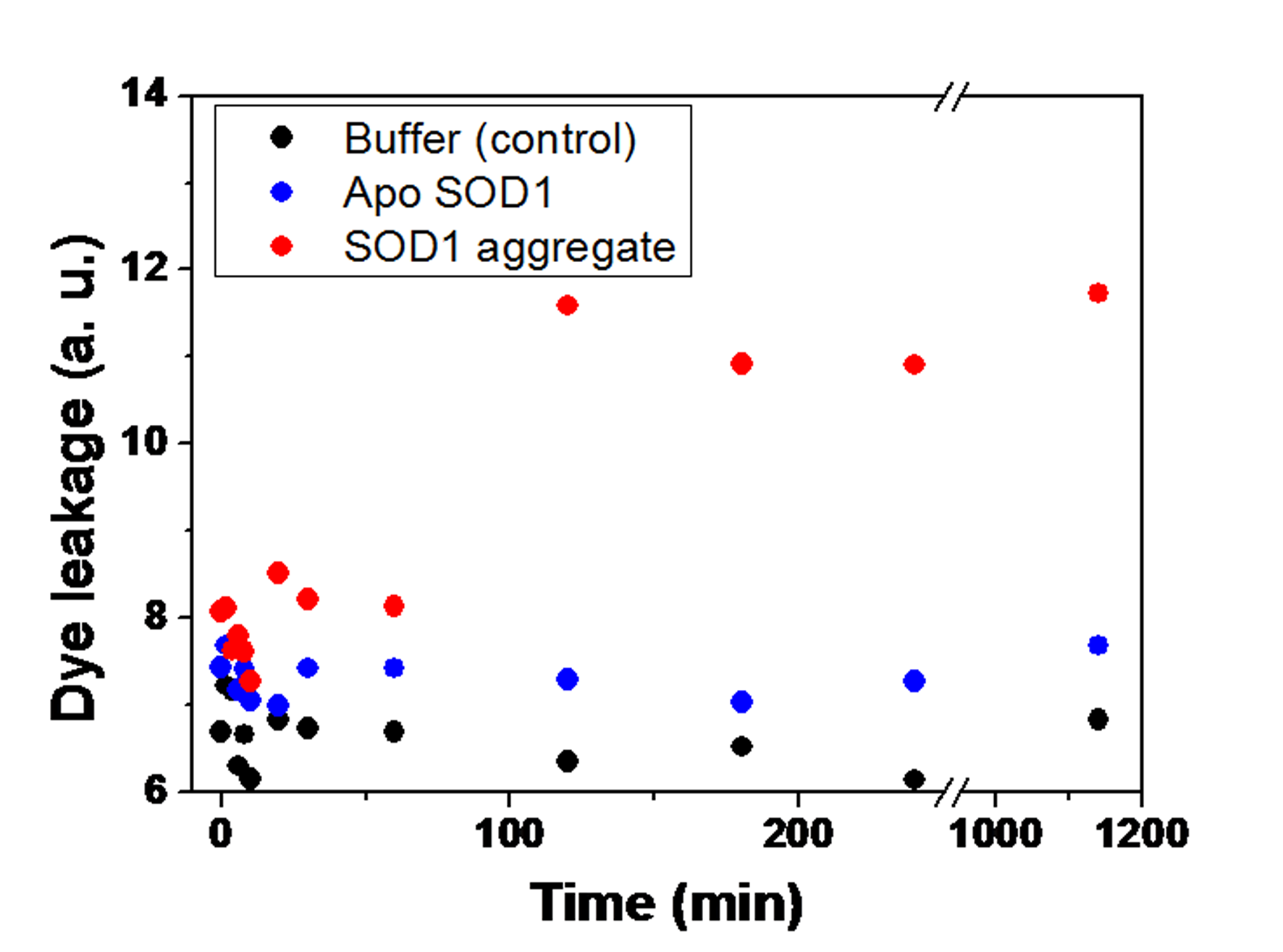

Supplement: Figure S6 — Dye leakage assay from the lipid vesicles induced by interactions between the lipid vesicles and SOD1 aggregates. (TIF) [file pone.0028982.s006.tif]

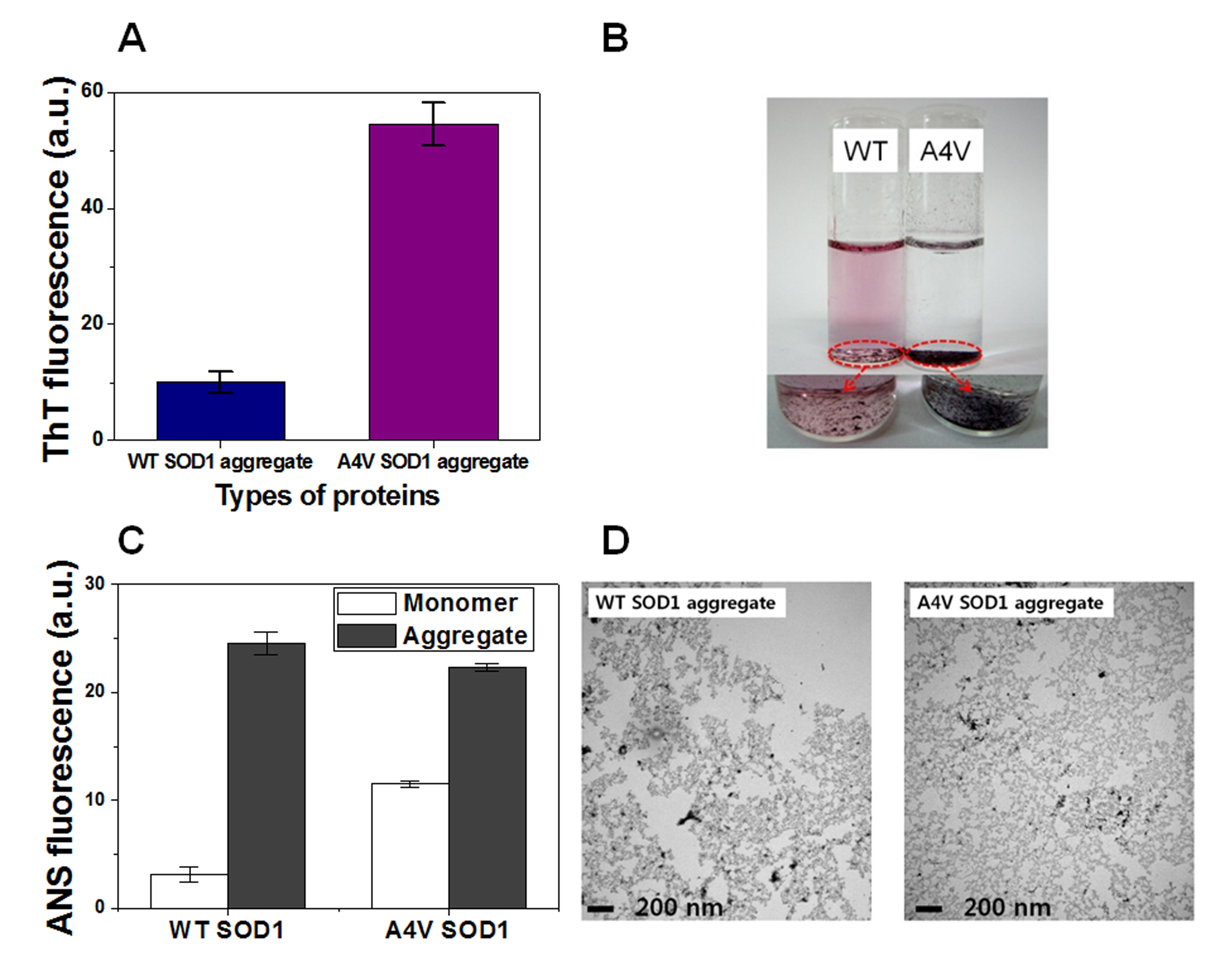

Supplement: Figure S7 — The A4V and WT SOD1 aggregates used in this study share a common morphology, but the kinetics of their aggregation is dramatically different. (A) ThT florescent intensity of the prepared SOD1 aggregates under same conditions (i.e., same protein concentration and destabilizing condition). (B) Visualization of the aggregates by formation of the precipitation, which are induced by gold nanoparticle binding to the SOD1 aggregates; The A4V aggregate is much more formed that of WT SOD1 under the same conditions (Note that the aggregation kinetic of A4V is faster than that of WT.). (C) and (D) ANS fluorescence enhancement and morphologies of prepared aggregates show the similarity between WT (left) and A4V (right). (TIF) [file pone.0028982.s007.tif]
